# Genetic Deep Convolutional Autoencoder Applied for Generative Continuous Arterial Blood Pressure via Photoplethysmography

**DOI:** 10.3390/s20143829

**Published:** 2020-07-09

**Authors:** Muammar Sadrawi, Yin-Tsong Lin, Chien-Hung Lin, Bhekumuzi Mathunjwa, Shou-Zen Fan, Maysam F. Abbod, Jiann-Shing Shieh

**Affiliations:** 1 Department of Mechanical Engineering, Yuan Ze University, Taoyuan 32003, Taiwan; muammarsadrawi@yahoo.com (M.S); mathunjwabhekie@gmail.com (B.M.); 2 AI R&D Department, New Era AI Robotic Inc., Taipei 105, Taiwan; lotusytlin@neweraai.com (Y.-T.L.); lance_lin@neweraai.com (C.-H.L.); 3 Department of Anesthesiology, College of Medicine, National Taiwan University, Taipei 100, Taiwan; shouzen@gmail.com; 4 Department of Electronic and Computer Engineering, Brunel University London, Uxbridge UB8 3PH, UK; Maysam.Abbod@brunel.ac.uk

**Keywords:** photoplethysmography, continuous arterial blood pressure, systolic blood pressure, diastolic blood pressure, deep convolutional autoencoder, genetic algorithm

## Abstract

Hypertension affects a huge number of people around the world. It also has a great contribution to cardiovascular- and renal-related diseases. This study investigates the ability of a deep convolutional autoencoder (DCAE) to generate continuous arterial blood pressure (ABP) by only utilizing photoplethysmography (PPG). A total of 18 patients are utilized. LeNet-5- and U-Net-based DCAEs, respectively abbreviated LDCAE and UDCAE, are compared to the MP60 IntelliVue Patient Monitor, as the gold standard. Moreover, in order to investigate the data generalization, the cross-validation (CV) method is conducted. The results show that the UDCAE provides superior results in producing the systolic blood pressure (SBP) estimation. Meanwhile, the LDCAE gives a slightly better result for the diastolic blood pressure (DBP) prediction. Finally, the genetic algorithm-based optimization deep convolutional autoencoder (GDCAE) is further administered to optimize the ensemble of the CV models. The results reveal that the GDCAE is superior to either the LDCAE or UDCAE. In conclusion, this study exhibits that systolic blood pressure (SBP) and diastolic blood pressure (DBP) can also be accurately achieved by only utilizing a single PPG signal.

## 1. Introduction

Blood pressure (BP) is the pressure driven by the blood circulation to the artery wall. Meanwhile, hypertension or high blood pressure (HBP) is an excessive amount of a given force against blood vessels. In addition, according to World Health Organization (WHO), HBP affects more than one billion people in the world [1].

With having an impact on many people, HBP can incite several diseases. It has a solid contribution to cardiovascular and renal diseases [2]. HBP also contributes to stroke and ischemic heart diseases [3]. Furthermore, HBP can generate vascular damage of the retina related to cardiovascular-based fatality [4]. These aforementioned studies make HBP-related inspection become significant.

Photoplethysmography (PPG), one of the vital signs, has been a solid indicator for some medical-related investigations. PPG has been deployed as the heart rate measurement in motion artifact-interfered conditions with an empirical mode decomposition-based filter and time-frequency evaluation [5]. It also has been utilized, alongside electrocardiography, for atrial fibrillation in acute stroke patients [6]. Another study involved the PPG morphological feature for hypertension early identification [7]. Moreover, Phillips et al. applied PPG sensors to non-invasively evaluate hemoglobin concentration [8]. Meanwhile, Perpetuini et al. supervised a general linear model-based PPG to evaluate the ankle–brachial index, which was initially measured using a commercial instrument as the gold standard [9]. In a recent study, entropy-based PPG evaluations have been successfully applied to distinguish between healthy and diabetic patients [10].

There are many previous studies that effectively demonstrate the substantial interconnection between PPG and BP. A study investigated the relationship of PPG with intermittent systolic and diastolic blood pressures using multi-scale entropy and ensemble neural network [11]. Sideris et al. evaluated continuous arterial blood pressure (ABP) using long short-term memory (LSTM) from patients in an intensive care unit (ICU) using only a PPG signal [12]. Furthermore, the hybrid of LSTM and artificial neural network (ANN) was performed via ECG and PPG to measure BP [13]. In addition, autoregressive moving average to investigate the blood pressure also by using features of the PPG signal related to the specific breathing conditions was performed and showed a quality evaluation [14]. Another study used multiple signals from ECG and PPG, and ballistocardiograms (BCG) were used to investigate systolic blood pressure (SBP) and diastolic blood pressure (DBP) by utilizing hybrid artificial intelligence (AI) methods [15]. Meanwhile, Slapničar et al. utilized 510 subjects of a single PPG signal with a ResNet deep learning model [16].

Generally, AI has been widely used in many fields. It has been used simultaneously with computational fluid dynamics in order to optimize the control scheme by adjusting the triangular membership function for the cooling system in a heat exchanger [17]. A hybrid AI, combining the extreme learning machine with the cuckoo search algorithm, was applied for biodiesel production [18]. Meanwhile, a study used neural network with the multi-armed bandit algorithm for solid oxide fuel cell problems [19]. Moreover, Zaidan et al. applied an AI-based model for gas turbine engine inspection [20].

Specific to medical-related studies, AI was utilized for detecting the depth of anesthesia, involving multi-vital signs [21]. Another study applied entropy-based calculation to extract the feature from one vital sign, which is the EEG. Meanwhile, the 5-s intermittent data from other vital signs were later combined with the extracted entropy value from the EEG. A wearable device-related study also utilized ANN in classifying arrhythmia [22]. Fast Fourier transform (FFT) was also administered to evaluate arrhythmia in the frequency domain. Moreover, the ANN model was also implemented to predict pneumonia [23,24].

Besides being widely utilized, AI has generalization problems [21]. The ensemble technique is likely used to help the model to deal with this difficulty and increase the accuracy of the models. However, selecting all the models for the ensemble system has not always been the best solution [25]. The combination of fuzzy clustering, ANN and the genetic algorithm (GA) was administered for the ensemble model for highly unbalanced data evaluation in emergency medical services [26]. The GA was called to investigate which models should be allocated to have a good ensemble system. Furthermore, this related study examined the quality of the model based on the area under the curve (AUC) from the receiver operating characteristic (ROC) as the fitness function. The result from this study [26] was convincingly supported by the study by Zhou et al. [25]. The ensemble model will definitely increase the accuracy of the result. Nevertheless, selecting several classifiers is likely to produce a better result than combining all of them [25].

Recently, ANN algorithms are moving towards a deeper structure, called deep neural network [27]. This system has been administered to substantial studies. Other methods, such as the convolutional neural network (CNN), have been used to predict arrhythmia with a very precise result with reference to a cardiologist [28]. Another powerful evidence by the CNN-based evaluation technique has also been performed to solve the seizure problem using encephalograms (EEG) [29]. Moreover, a study to evaluate the depth of anesthesia that utilized short-time Fourier transform (STFT) and CNN [30] was investigated to evaluate a four-class system classification in anesthesia from this related study in comparison with several CNN models.

As revealed in the aforementioned details, PPG, as one of the vital signs, is highly potentially able to estimate the blood pressure system. Further, the AI method, especially the deep neural network, has been very widely utilized in many areas particularly medical-related fields either in the classification or the regression system. Moreover, with the help of the GA, as the optimizer, the ensemble model of the deep learning algorithm is prospectively utilized. Hence, the aim of this paper is to investigate generative continuous ABP using deep neural network models via a deep convolutional autoencoder (DCAE) by utilizing only a single PPG sensor. Finally, the GA will form the ensemble model from the evaluation of the cross-validation models.

## 2. Materials and Methods

This study has been approved by the Research Ethics Committee, National Taiwan University Hospital (NTUH) in Taiwan. Furthermore, written informed consent was received for permission by the patients. In total, a dataset of 18 patients during surgical operation was used for the evaluation. The dataset was acquired using an MP60 IntelliVue Patient Monitor (Koninklijke Philips N.V, Amsterdam, Netherlands) that is connected to a PC. More detail about the data collection can be seen on a study conducted by Liu et al. [31].

Regarding the dataset and the deep learning evaluations, the sampling rate of the PPG and ABP is 128 Hz. The window size evaluation was based on each 5-s signal, both the PPG and ABP. This phenomenon means that each 5-s PPG signal is able to predict the corresponding 5-s of the ABP signal. Initial total data were 42,498 sequences of 5-s windows of PPG and ABP. The data were manually filtered based on their signal quality due to the diathermy effect or nurse activities. Manual filtration was performed by eye by evaluating if either the PPG signal or the ABP signal was noisy. The evaluation was based on the PPG and ABP signal shapes. Finally, the abnormal sequences of these 5-s signals were discarded. This reduced the data amount by about 14% to 36,516 sequences. In this study, the range of the data was limited between 10 and 250 mmHg. Some noisy ABP signals were likely affected by the high-frequency noise. The dataset was randomly divided into 85% and 15% respectively for the training and testing data. MATLAB R2014b (The MathWorks, Inc., Natick, Massachusetts, USA) was utilized for pre-processing the data and post-processing the results. TensorFlow (Ver. 1.15.2) [32] and Keras (Ver. 2.3.1) were utilized in Google Colaboratory (Google Inc., California, USA) for the deep learning training using Python 3.6. The training was conducted for 200 epochs with a batch size of 16 with Adam optimizer [33]. The model checkpoint was also set for the training system. Further, the training data were shuffled. Finally, the cross-validation (CV) method was conducted to investigate the model regularity.

The evaluations were conducted based on mean absolute error (MAE), root mean squared error (RMSE), and Pearson’s linear correlation coefficient. Furthermore, the Bland–Altman plot model was provided for comparison purposes. These evaluations are given in Equations (1-3). The Pearson’s linear correlation coefficient evaluates between the MP60, as the gold standard, and the generated continuous arterial blood pressures. It also investigates the systolic blood pressure (SBP) and diastolic blood pressure (DBP) values, by taking the maximum and minimum values from the continuous signal, respectively for SBP and DBP, between the MP60 IntelliVue Patient Monitor and the models. The given error is in mmHg. The $R_{x,y}$ value is in range between 0 and 1. The model and the reference are perfectly correlated when the given $R_{x,y}$ value equals 1.

(1) MAE=1n∑i=1n|xi−yi|

(2) RMSE=1n∑i=1n(xi−yi)2

(3) Rx,y=∑i=1n(xi−x¯)(yi−y¯)[∑i=1n(xi−x¯)2∑i=1n(yi−y¯)2]12

where $x_{i}$ is the reference, $y_{i}$ is the estimated result, n is the number of samples, $\bar{x}$ is the mean of the reference, and $\bar{y}$ is the mean of the predicted result.

**Figure A1:**
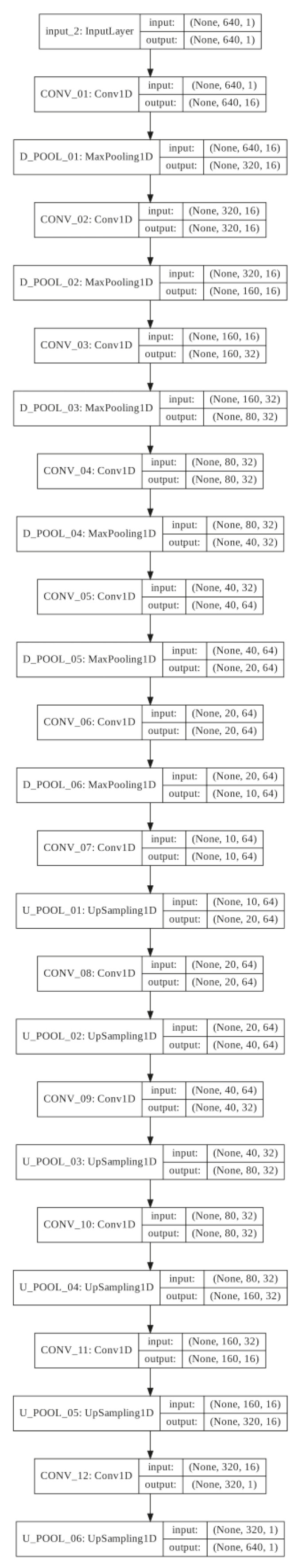
LeNet-5 based deep convolution autoencoder (LDCAE) structure.

This study evaluates two DCAE models. Basically, the autoencoder structure has the latent space between the input and the output layers. The first model is generated based on the LeNet-5 CNN model [34]. Originally, this model worked for the digit recognition system. The architecture of this model is relatively simple compared with other models. The convolution layer in this model is regularly followed by subsampling. For the classification system, there are several fully connected layers installed to the network. This study uses only the convolution layer with the subsampling from the original LeNet-5 model to form the encoder. Meanwhile, the decoder utilizes the opposite way of the encoder. The summary of the LeNet-5-based deep convolutional autoencoder (LDCAE) utilized in this study can be seen in Figure A1 in Appendix A. From this figure, it can be seen that the original 5 s of the one-dimensional PPG signal and the sampling rate of 128 Hz, with a size of 640 points, are used for the input layer. For the encoder, this study applies an increasing filter size. All convolution layers administer the rectified linear unit (ReLU) activation function, shown in Equation (4). This structure also uses the same padding. After the input layer, for the encoder, the first convolution layer starts with 16 filters and ends with 64 filters. However, the decoder works with initially 64 filters to 16 filters. The output layer is equal to the input layer. This layer is the 5-s ABP signal. This model has equal total parameters and trainable parameters, which total about sixty thousand parameters.

f(X) = max(0,X)(4)

where X is the input signal.

**Figure A2:**
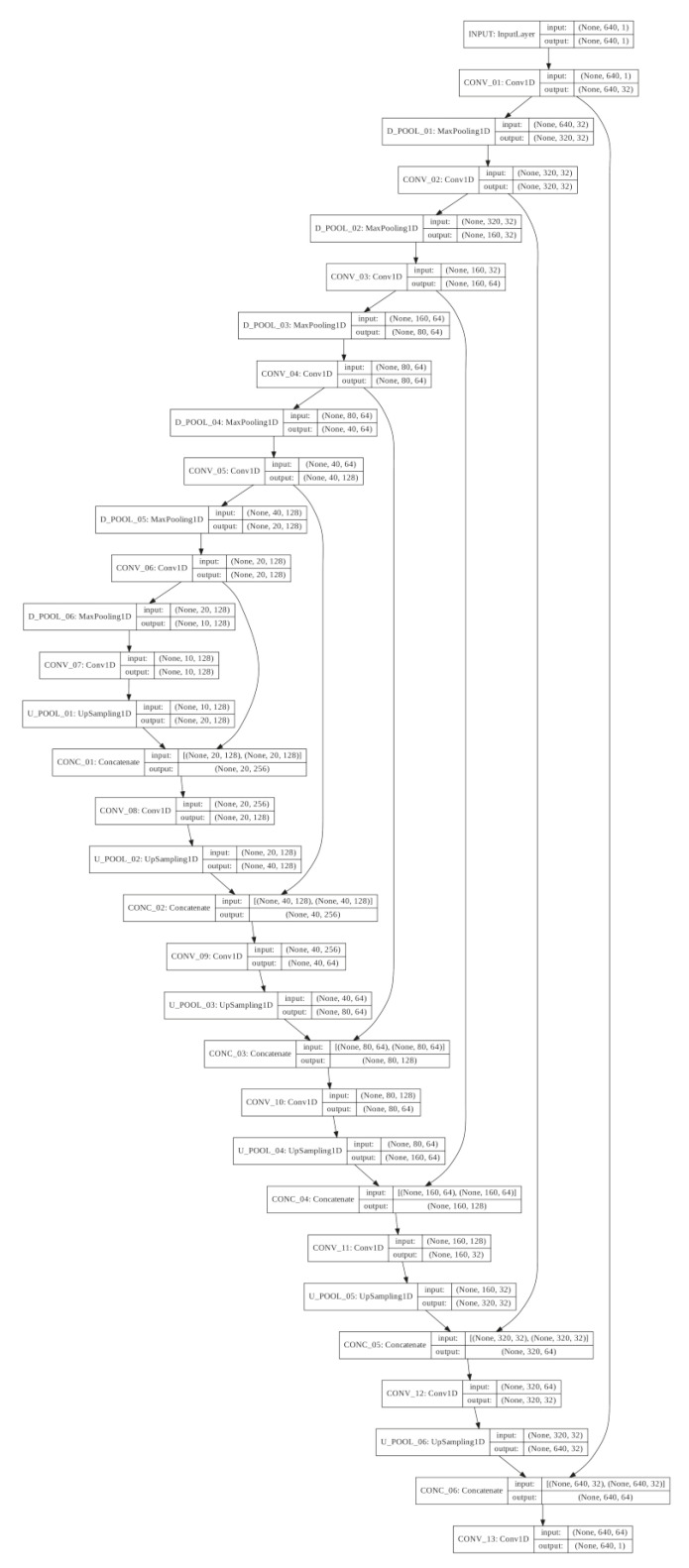
U-Net based deep convolution autoencoder (UDCAE) structure.

Another model is the deep convolutional autoencoder based on the U-Net architecture [35]. This model was originally applied for biomedical segmentation. One of the reasons behind the uniqueness of the U-Net model is the concatenating between a layer in the encoder and another layer in the decoder that has the same feature map. The detailed structure of the U-Net-based deep convolutional autoencoder (UDCAE) used in this study is shown in Figure A2 in Appendix A. In parallel with the LDCAE model, this model also has an input size of 640 data points of the PPG. The encoder and decoder structures are also very identical to the LDCAE. However, the first filter in the encoder has 32 filters and ends with 256 filters. Further, the concatenated layer filters in the decoder are formed by considering the filter from the encoder layer. The UDCAE also utilizes the ReLU activation function. This UDCAE model has an equal total number of settings and trainable parameters, which total about three hundred thousand parameters. These numbers of parameters are much bigger compared with the LDCAE structure.

Moreover, a 10-fold cross-validation (CV) system is conducted to evaluate the data generalization to the models. This CV method uses a leave-testing-out cross validation technique, meaning that the CV model shuffles only the training part and keeps the testing data outside the shuffling system. The highest average BP of the CV fold, combining the DCAE models, is selected as the best single model.

Finally, this study deploys genetic algorithm (GA) optimization, named the genetic deep convolutional autoencoder (GDCAE), to ensemble the ten CV models for each LDCAE and UDCAE. Each CV model has equally distributed weights, meaning each model will have the chance to be combined with other models. Therefore, the GA will have a total of 20 bits for each chromosome. The chromosomes are encoded in 32 bits binary format. Zero means the model is not selected and one means the model is selected. The GA is set with a single point crossover, 95% mutation rate and 2000 generations. The fitness function is given by Equation (5). This equation is a modified version of Equation (3). Specifically, Equation (5) calculates the average Pearson’s linear correlation coefficient between SBP and DBP, meaning that the weights are equally distributed.

(5) Rbp¯=12(∑i=1n(xi,sbp−xsbp¯)(yi,sbp−ysbp¯)[∑i=1n(xi,sbp−xsbp¯)2∑i=1n(yi,sbp−ysbp¯)2]12+∑i=1n(xi,dbp−xdbp¯)(yi,dbp−ydbp¯)[∑i=1n(xi,dbp−xdbp¯)2∑i=1n(yi,dbp−ydbp¯)2]12)

## 3. Results

This study utilizes deep convolutional autoencoder (DCAE) models to generate the continuous arterial blood pressure signal (ABP) by using single photoplethysmography (PPG). The results produced by the models are compared to investigate the better model compared to the MP60 IntelliVue Patient Monitor as the gold standard. The evaluations cover the continuous arterial blood pressure signal with systolic and diastolic blood pressures.

**Figure 1:**
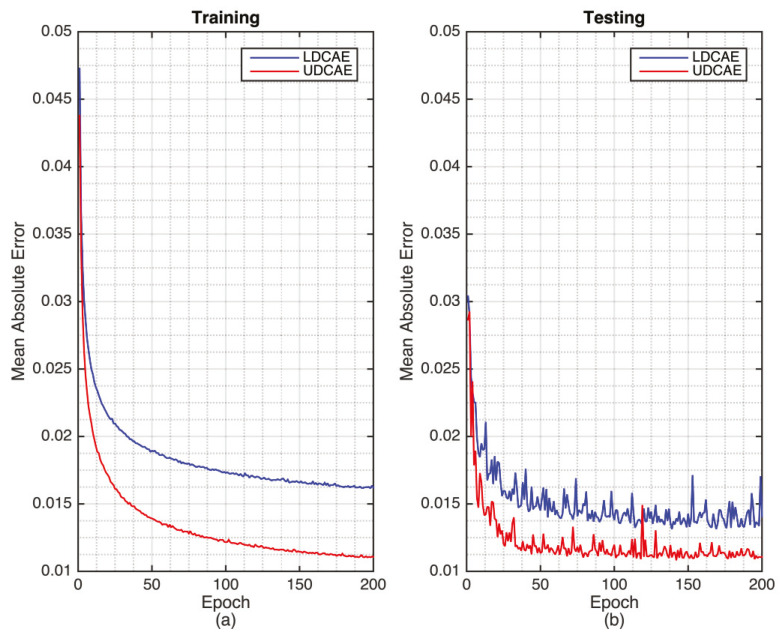
The training (**a**) and testing (**b**) of the LeNet-5 based deep convolution autoencoder (LDCAE) and U-Net-based deep convolutional autoencoder (UDCAE) models.

The training of the DCAE models can be seen in Figure 1 where UDCAE converges faster and better than the LDCAE model. Furthermore, for the testing phase, the UDCAE model also provides a preferable result compared with the LDCAE. In addition, the UDCAE model shows relatively less fluctuation.

**Figure 2:**
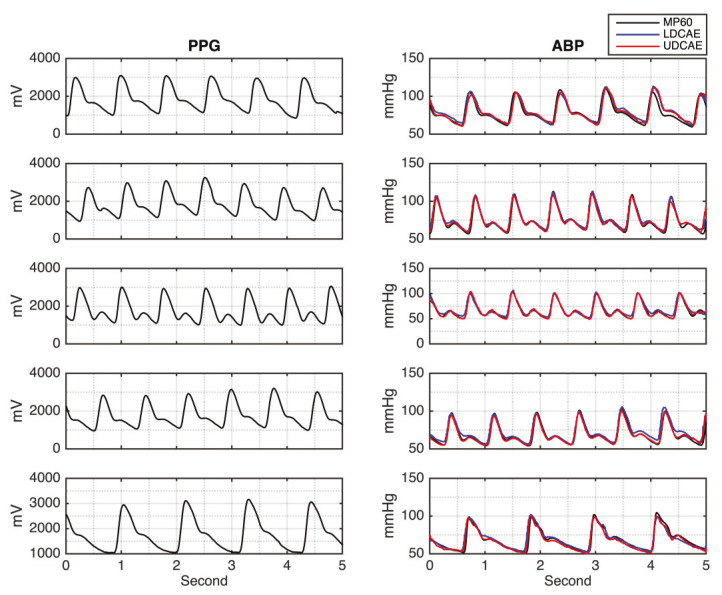
The photoplethysmography (PPG) input signal and arterial blood pressure (ABP) results between LDCAE and UDCAE models in comparison to MP60 IntelliVue Patient Monitor.

Figure 2 shows the input of the PPG signal and its corresponding output of the continuous ABP signals, generated by the DCAE-based models for the testing results. It can be seen that both models, LDCAE and UDCAE, successfully produce continuous ABP. In addition, Figure 2 also reveals that SBP and DBP can be accurately estimated. Both models display a fine estimation result in that the PPG has either a significant or non-significant second peak.

**Figure 3:**
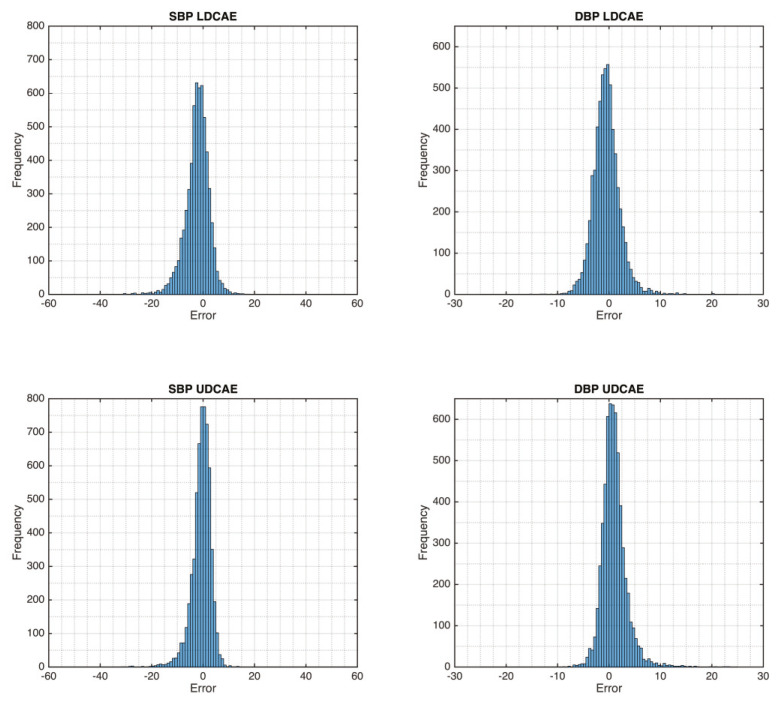
The error comparison between DCAE-based models and MP60 IntelliVue Patient Monitor.

After performing the continuous ABP, the evaluation of SBP and DBP is further investigated. The maximum value of a 5-s segment is defined as SBP. Meanwhile, the minimum value is DBP. This approach is deployed for both the DCAE models and the MP60, as the gold standard. The evaluation of SBP and DBP can be seen on the error distribution graphs shown in Figure 3. From this figure, both LDCAE and UDCAE are compared to the MP60 IntelliVue Patient Monitor values. It can be seen that the UDCAE model produces a better outcome by delivering a higher frequency of results approaching zero than the LDCAE model.

**Figure 4:**
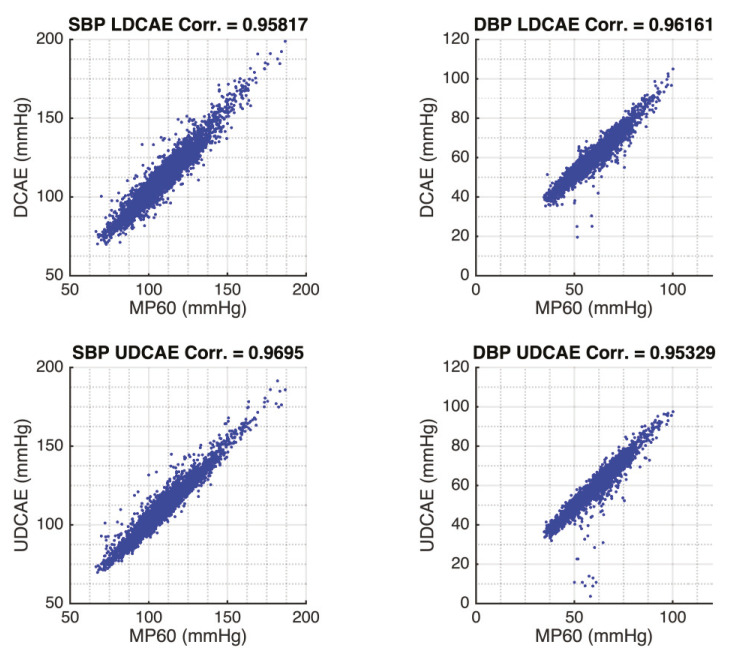
The Pearson’s linear correlation comparison between DCAE-based models and MP60 IntelliVue Patient Monitor.

Furthermore, to investigate the model prediction accuracy of SBP and DBP, the results are compared to the MP60 using Pearson’s linear correlation coefficient, which shows heterogeneous outcomes. The UDCAE has a slightly better result in the SBP prediction. Meanwhile, the LDCAE displays insignificantly better results for the DBP estimation. The detailed evaluation is shown in Figure 4.

**Figure 5:**
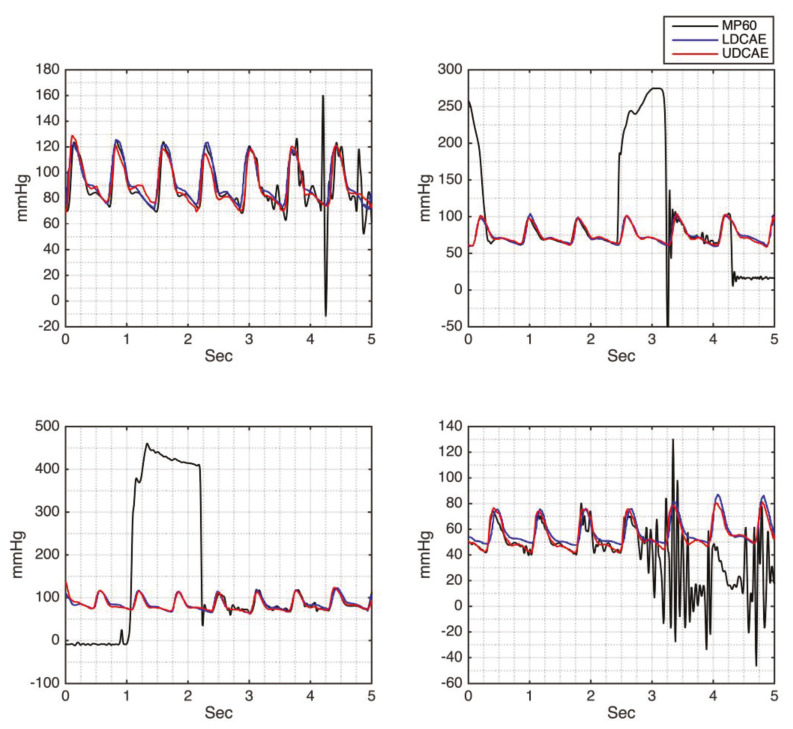
The comparison of the noisy MP60 ABP signal and the generated ABP signal by the LDCAE and UDCAE models.

Another powerful approach given by the DCAE models is the ability to generate a continuous ABP signal that is not interfered by any noise since a good-quality PPG is supplied. From Figure 5, it can be seen that some signals produced by the MP60 IntelliVue Patient Monitor are relatively noisy. However, this has been overcome by the DCAE models. Moreover, the predicted SBP and DBP values are comparable, by comparing them to either the preceding or the succeeding cycles.

Cross-validation is later performed in order to evaluate the data generalization and ensemble combination. The results show that the data have very high generalization. Good generalization is given by the standard deviation of the Pearson’s linear correlation for SBP, DBP and the waveform evaluations, given in Table 1. Moreover, the relatively small standard deviation of RMSE and MAE for SBP, DBP and the waveform error evaluations are shown in Table 2.

The selection of the best single model from the CV results is evaluated based on Pearson’s linear correlation coefficient given in Table 1. It can be seen that the fourth CV model provides the highest average value between SBP and DBP, which is 0.9643. Hence, this model is selected as the best single model.

After having the CV models, both from LDACE and UDCAE, the genetic algorithm-based optimization deep convolutional autoencoder (GDCAE) is subsequently performed. The GA will work as the selector of the DCAE models that will be combined for the ensemble system. As the result, the CV models 1, 2, 3, 4, 5 and 10 are selected by the GA from the LDCAE model. Meanwhile, GA selects all the UDCAE models, except the first model. The results also show the reliability of the fourth model of the LDCAE and UDCAE systems.

**Figure 6:**
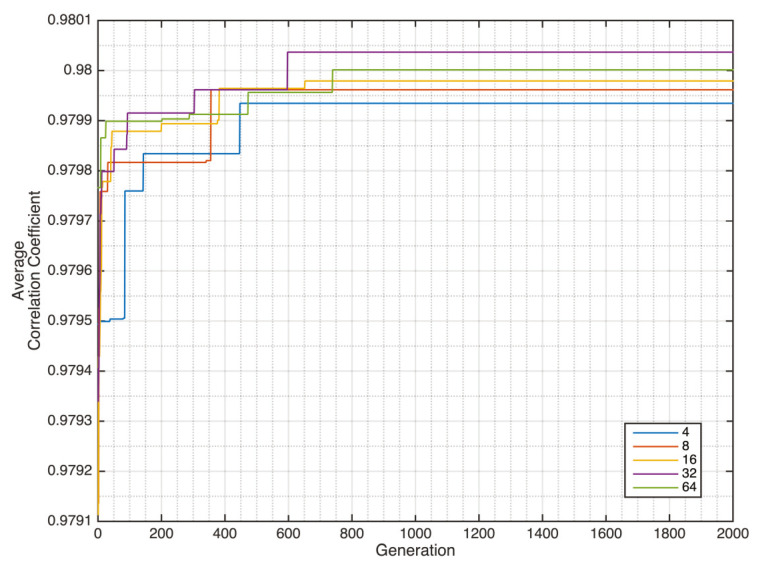
Genetic deep autoencoder (GDCAE) generation convergence.

The convergence of the GDCAE is shown in Figure 6. Several chromosome sizes of 4, 8, 16, 32 and 64 are investigated. The average result from the SBP and DBP of GDCAE is 0.98004. This GDCAE result is better compared with the average value of SBP and DBP from the best single CV model, 0.960 and 0.961 for the LDCAE and UDCAE models, respectively. By having this combination, the GA-optimized reconstructed signal is later performed. The results also provide some improvements in comparison with the best CV model in Pearson’s linear correlation coefficient and error evaluations, which can be seen in Table 3.

**Figure 7:**
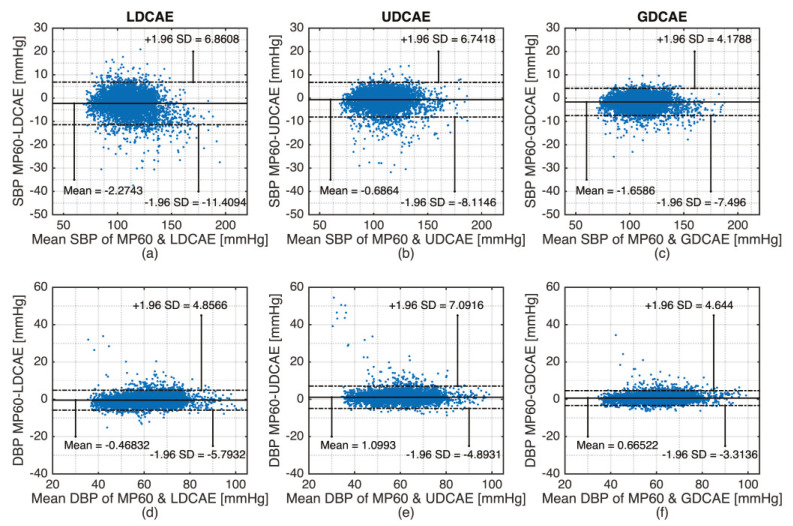
Bland–Altman plot. (**a**) LDCAE systolic blood pressure (SBP); (**b**) UDCAE SBP; (**c**) GDCAE SBP; (**d**) LDCAE diastolic blood pressure (DBP); (**e**) UDCAE DBP; (**f**) GDCAE DBP.

Furthermore, the Bland–Altman evaluation results can be seen in Table 4 and Figure 7. Even though the GDCAE has a slightly inferior result for the mean value to the LDCAE and UDCAE respectively for DBP and SBP, the GDCAE has lower standard deviation compared with other models. Furthermore, for GDCAE, the 95% confidence band, ± 1.96 of standard deviation of the difference, produces smaller distances compared with LDCAE and UDCAE both for SBP and DBP. Qualitative results are shown in Figure 7, which is a good indication that the GDCAE model provides better prediction results between the 95% confidence band compared with the LDCAE and UDCAE models.

## 4. Discussion

Initially in this study, the PPG signal is trained by using DCAE models, LeNet-5- and U-Net-based models, to generate a continuous arterial blood pressure (ABP) signal. In this step, the PPG- and MP60 IntelliVue Patient Monitor-generated continuous arterial blood pressure signals are compared. Moreover, systolic and diastolic blood pressures are evaluated by root mean squared error (RMSE), mean absolute error (MAE) and the Pearson’s linear correlation coefficient between the models with the MP60 IntelliVue Patient Monitor as the gold standard. Finally, the GA-regulated DCAE based on the cross-validation results is deployed to ensemble the model and evaluate the system.

In order to investigate the quality of the proposed methods, a comparative study to the previously organized research was conducted. The comparison method included the dataset, input signal, methodology, generative system, error evaluations and linear correlations. The details of the comparative studies are given in Table 5. Sideris et al. [12] utilized the forty-two-patient dataset from MIMIC PhysioNet, originally a two hundred-patient dataset, after applying some filtering steps based on the quality of the blood pressure signal. This study also only used a single PPG signal. The overlapped window size was used in order to form either the training or testing data. Further, LSTM, one of the deep neural network methods, was applied for the prediction. One of the essential achievements from this study is the ability to generate a continuous arterial blood pressure signal. As it can be seen, the capability of LSTM is able to produce continuous arterial blood pressure by only utilizing the PPG signal. However, it did not mention specifically about the RMSE of the DBP. Nevertheless, in this study, they provided a table consisting of the tabulated RMSE result of SBP, DBP and ABP. With full respect to all the authors in this study [12], we re-evaluate the ABP and SBP results based on the corresponding table. This is conducted to recalculate the mean and standard deviation, which were found to have very identical results to their reported results. Hence, we perform the DBP calculation, in parallel to the aforementioned method for the ABP and SBP calculations. The results of DBP, for mean and standard deviation, are 1.98±1.06 mmHg. In comparison with our study, this study has slightly better results in the RMSEs of SBP and DBP error evaluations. However, in this study, the GDCAE provides a better outcome in the waveform error evaluation, which is 0.984. Moreover, our GDCAE also delivers a superior solution for the correlation coefficient for the waveform evaluation. Meanwhile, Sideris et al. [12] did not provide any information about the SBP and DBP correlation coefficient results.

Another study related to blood pressure evaluation was conducted by Tanveer et al. [13]. This study applied multiple vital signs, which are ECG and PPG. This study used the dataset of thirty-nine patients, from originally ninety-three patients, of the MIMIC I PhysioNet database. This study had 16-s and 40-s window sizes, with 125 Hz of sampling frequency. This study also deployed the LSTM method, similar to the study performed by Sideris et al. [12], alongside the ANN. This study provided an outstanding result in the error estimation in mmHg. Based on the combination of LSTM and ANN methods, their study produced significantly small RMSEs, which are 1.26 mmHg and 0.73 mmHg, respectively for SBP and DBP. Moreover, the MAEs for SBP and DBP are respectively 0.93 mmHg and 0.52 mmHg. Identical to the error evaluation, the Pearson’s linear correlation coefficient evaluation is also an exceptional finding. Nearly perfectly correlated systems are produced, which are 0.999 and 0.998 for SBP and DBP, respectively. This result is produced by the longer size, which is the 40-s window size system. However, this method has a drawback. It did not provide the information about generative continuous arterial blood pressure.

A study investigated by Zadi et al. [14] used fifteen young subjects. This study evaluated the blood pressure based on two conditions, which are normal breath and breath hold. The autoregressive moving average (ARMA) was deployed in the modeling. This study produced a relatively good result. It has RMSEs of 7.21 and 5.12 mmHg, respectively for systolic and diastolic blood pressure. However, neither correlation coefficient for waveform, SBP nor DBP was provided. Moreover, there was no available generative continuous ABP signal investigation.

Another comparative study is the finding by Eom et al. [15]. This study was conducted on fifteen subjects. It used several vital signs, which are ECG, PPG and BCG. The 5-s window size was also used in this study. The combination of CNN, bidirectional gated recurrent unit (Bi-GRU) and attention mechanism. The result showed the produced MAEs and standard deviations are 4.06 ± 4.04 and 3.33 ± 3.42 mmHg, respectively for SBP and DBP. However, this study has a disadvantage, which is no generative continuous blood pressure estimation was performed.

The latest study conducted by Slapničar et al. [16] utilizing 510 subjects using a single PPG with a ResNet-based model is used. The results showed 9.43 and 6.88 mmHg of MAE respectively for SBP and DBP. Nevertheless, there is no given information about generative continuous arterial blood pressure evaluation.

As it can be seen from the aforementioned information comparing our proposed methods to previously performed studies, our study shows assorted advantages. Our proposed methods, working based on the deep autoencoder and using only a single PPG signal, provide a leading achievement for the correlation coefficient for the waveform of the generative continuous blood pressure signal. Additionally, our proposed methods produce highly correlated results of the estimated SBP and DBP to the MP60 IntelliVue Patient Monitor, as the gold standard.

**Figure 8:**
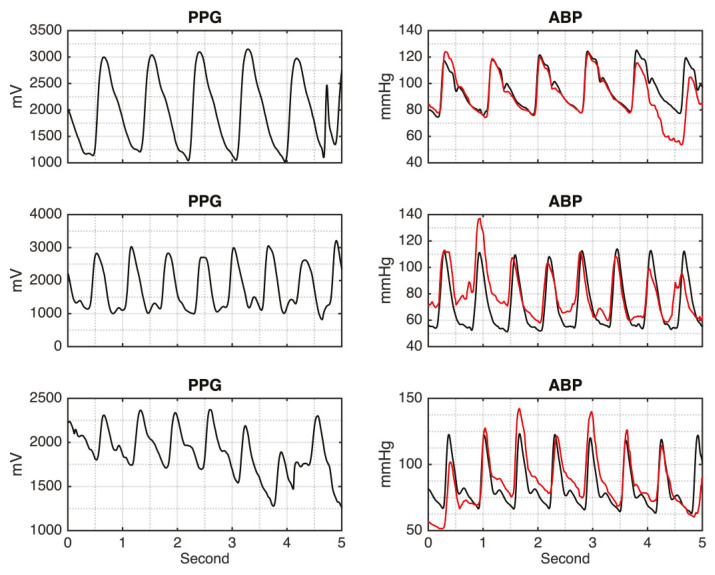
Low-quality generated continuous ABP result.

However, this study has several limitations. The number of the patients utilized in this study is relatively small. In addition, most of the utilized patient data are during surgery. This unconscious condition may reduce the noise interfering the PPG signal, especially for the motion artifact. For this reason, automatic-based filters should be applied in future work for conscious subjects. Furthermore, the algorithm to evaluate SBP and the DBP from a 5-s sliding window can be improved. This technique is selected based on the consideration that either SBP or DBP do not fluctuate significantly within five seconds. Furthermore, more advanced statistical analysis can be applied. In addition, the noisy PPG signal can contribute to the low-quality continuous ABP prediction, as it can be seen in Figure 8.

## 5. Conclusions

This study demonstrates that deep convolutional autoencoder methods with GA-based optimization have successfully evaluated the continuous arterial blood pressure system by only using a single PPG signal. In addition, supporting the previous studies, this study also shows straightforward information that the PPG is highly correlated with continuous arterial blood pressure. Hence, the SBP and DBP measurements can be precisely achieved by only using a single PPG signal.

## Figures and Tables

**Table 1:** The Pearson’s Linear Correlation Coefficient Evaluation of LDCAE and UDCAE Models from the Cross-Validation (CV) Method. Note: bold value is the best single CV model.

CV	Correlation Coefficient						
	SBP		DBP		Waveform		Average
	LDCAE	UDCAE	LDCAE	UDCAE	LDCAE	UDCAE	
1	0.956	0.958	0.958	0.953	0.968	0.974	0.9612
2	0.960	0.961	0.954	0.942	0.969	0.974	0.9600
3	0.962	0.965	0.951	0.941	0.968	0.975	0.9603
**4**	**0.958**	**0.969**	**0.962**	**0.953**	**0.968**	**0.976**	**0.9643**
5	0.954	0.964	0.963	0.962	0.966	0.975	0.9640
6	0.951	0.960	0.959	0.956	0.966	0.974	0.9610
7	0.956	0.957	0.947	0.951	0.967	0.973	0.9585
8	0.959	0.964	0.949	0.956	0.968	0.976	0.9620
9	0.957	0.963	0.947	0.946	0.966	0.975	0.9590
10	0.958	0.968	0.963	0.947	0.967	0.975	0.9630
Mean	0.957	0.963	0.955	0.951	0.967	0.975	
STD	0.003	0.004	0.007	0.007	0.001	0.001	

**Table 2:** Error Evaluations of SBP and DBP from LDCAE and UDCAE Models.

CV	SBP				DBP			
	LDCAE		UDCAE		LDCAE		UDCAE	
	RMSE	MAE	RMSE	MAE	RMSE	MAE	RMSE	MAE
1	4.69	3.44	4.62	3.26	3.10	2.22	3.06	1.82
2	4.63	3.39	4.30	3.11	3.25	2.18	3.87	2.23
3	4.91	3.72	4.81	3.42	3.09	2.04	3.38	1.92
4	5.19	3.80	3.85	2.73	2.76	2.00	3.25	1.95
5	5.11	3.64	4.12	3.01	2.70	1.86	3.01	1.96
6	6.48	4.85	5.11	3.47	2.86	2.03	3.02	1.78
7	5.02	3.61	4.48	3.07	3.40	2.14	3.25	1.99
8	4.88	3.57	4.54	3.14	3.26	2.12	3.17	1.90
9	4.63	3.39	5.18	3.65	3.27	2.08	3.30	1.77
10	6.39	4.93	5.12	3.71	2.79	1.04	3.29	1.82
Mean	5.19	3.83	4.61	3.26	3.05	1.97	3.26	1.91
STD	0.68	0.57	0.45	0.31	0.25	0.34	0.25	0.14

**Table 3:** Comparison between the LDCAE, UDCAE and GDCAE Models.

Method	Correlation Coefficient			Error [mmHg]		
	Waveform	SBP	DBP	Waveform	SBP	DBP
LDCAE	R = 0.968	R = 0.958	R = 0.962	RMSE = 5.10 MAE = 3.52	RMSE = 5.19 MAE = 3.80	RMSE = 2.76 MAE = 2.00
UDACE	R = 0.976	R = 0.969	R = 0.953	RMSE = 4.25 MAE = 2.77	RMSE = 3.85 MAE = 2.73	RMSE = 3.25 MAE = 1.95
GDCAE	R = 0.984	R = 0.981	R = 0.979	RMSE = 3.46 MAE = 2.33	RMSE = 3.41 MAE = 2.54	RMSE = 2.14 MAE = 1.48

**Table 4:** Bland–Altman DCAE Model Comparison.

Methods	Mean [mmHg]		STD [mmHg]		−1.96 STD [mmHg]		+1.96 STD [mmHg]	
	SBP	DBP	SBP	DBP	SBP	DBP	SBP	DBP
LDCAE	−2.274	−0.468	4.661	2.717	−11.410	−5.793	6.862	4.857
UDCAE	−0.686	1.099	3.790	3.057	−8.114	−4.893	6.742	7.091
GDCAE	−1.659	0.665	2.978	2.030	−7.496	−3.314	4.178	4.644

**Table 5:** Comparative Results for Dataset and Methodology Between the Proposed Method and Previous Related Studies.

Error [mmHg]	DBP	RMSE = 1.98 STD = 1.06	RMSE = 0.73 MAE = 0.52	RMSE = 5.12	MAE = 3.33 STD = 3.42	MAE = 6.88	RMSE = 2.76 MAE = 2.00	RMSE = 3.25 MAE = 1.95		RMSE = 2.14 MAE = 1.48	
	SBP	RMSE = 2.58 STD = 1.23	RMSE = 1.26 MAE = 0.93	RMSE = 7.21	MAE = 4.06 STD = 4.04	MAE = 9.43	RMSE = 5.19 MAE = 3.80	RMSE = 3.85 MAE = 2.73		RMSE = 3.41 MAE = 2.54	
	Waveform	RMSE = 6.04 STD = 3.26	N/A	N/A	N/A	N/A	RMSE = 5.10 MAE = 3.52	RMSE = 4.25 MAE = 2.77		RMSE = 3.46 MAE = 2.33	
Correlation Coefficient	DBP	N/A	0.998	N/A	R^2^ = 0.49	N/A	R = 0.962	R = 0.953		R = 0.979	
	SBP	N/A	0.999	N/A	R^2^ = 0.52	N/A	R = 0.958	R = 0.969		R = 0.981	
	Waveform	Mean = 0.95 STD = 0.045	N/A	N/A	N/A	N/A	R = 0.968	R = 0.976		R = 0.984	
Gen. Cont. ABP		Yes	No	No	No	No	Yes				
Method		LSTM	ANN + LSTM	ARMA	CNN + Bi-GRU + Attention	Spectro temporal ResNet	LDCAE		UDCAE		GDCAE
Input Signal		PPG	ECG + PPG	PPG	ECG + PPG + BCG	PPG	PPG				
Dataset		42 subjects, MIMIC PhysioNet	39 subjects, MIMIC PhysioNet	15 subjects	15 subjects	510 subjects, MIMIC III PhysioNet	18 subjects, NTUH, Taiwan				
Studies		Sideris et al. [12]	Tanveer et al. [13]	Zadi et al. [14]	Eom et al. [15]	Slapničar et al. [16]	Proposed
